# Solvothermal water-diethylene glycol synthesis of LiCoPO_4_ and effects of surface treatments on lithium battery performance†

**DOI:** 10.1039/c8ra08785g

**Published:** 2019-01-04

**Authors:** Min Zhang, Nuria Garcia-Araez, Andrew L. Hector, John R. Owen, Robert G. Palgrave, Michael G. Palmer, Samantha Soulé

**Affiliations:** School of Chemistry, University of Southampton Highfield Southampton SO17 1BJ UK A.L.Hector@soton.ac.uk; Department of Chemistry, University College London 20 Gordon Street London WC1H 0AJ UK

## Abstract

Olivine-structured LiCoPO_4_ is prepared *via* a facile solvothermal synthesis, using various ratios of water/diethylene glycol co-solvent, followed by thermal treatment under Ar, air, 5%H_2_/N_2_ or NH_3_. The diethylene glycol plays an important role in tailoring the particle size of LiCoPO_4_. It is found that using a ratio of water/diethylene glycol of 1 : 6 (v/v), LiCoPO_4_ is obtained with a homogenous particle size of ∼150 nm. The bare LiCoPO_4_ prepared after heating in Ar exhibits high initial discharge capacity of 147 mA h g^−1^ at 0.1C with capacity retention of 70% after 40 cycles. This is attributed to the enhanced electronic conductivity of LiCoPO_4_ due to the presence of Co_2_P after firing under Ar. The effects of carbon, TiN and RuO_2_ coating are also examined. Contrary to other studies, it is found that the solvothermally synthesised LiCoPO_4_ samples produced here do not require conductive coatings to achieve good performance.

## Abstract 2

Solvothermal water-diethylene glycol synthesis of LiCoPO_4_, followed by thermal treatment under Ar, air, 5%H_2_/N_2_ or NH_3_ was investigated.

## Graphical abstract

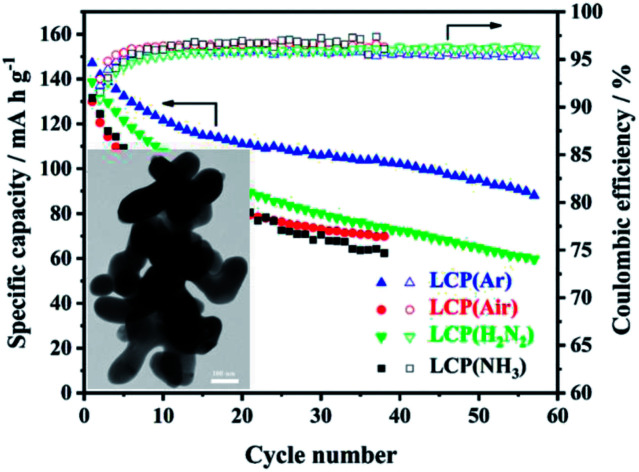

## Introduction

Development of energy storage and conversion devices is vital to address the increasing energy crisis and ecological concerns in the 21st century.^1^ Although a variety of renewable energy technologies such as solar cells, fuel cells and biofuels have been developed,^2–5^ the need for efficient, cheap and reliable storage devices is still pressing when using renewable energies.^5^ Electrical energy storage like lithium batteries and supercapacitors are effective strategies in making the energy output much cleaner.^6–8^ As one of the most efficient energy storage devices, lithium-ion batteries (LIBs) are used in portable electronic devices and large-scale electric vehicles^9–12^ due to their high energy density, high power density and light weight compared with conventional batteries.^13–15^ The olivine-structured LiMPO_4_ (M = Fe, Mn, Co, Ni) phases have been intensively investigated as cathode materials for LIBs,^15–18^ especially LiFePO_4_ which has been successfully commercialised.^19–24^ LiCoPO_4_ has also attracted significant attention due to its high redox potential (4.8 V *vs.* Li/Li^+^) and high theoretical capacity (167 mA h g^−1^), making it a promising future cathode material for high-voltage LIBs.^25–30^ However, use of LiCoPO_4_ as a cathode in practical applications has been hindered by its unsatisfactory cycle stability and rate capability, which could be mainly attributed to its low electronic conductivity^17,31–36^ and poor Li^+^ ionic conductivity^36–41^ relating to the one-dimensional ion transport channels,^42^ as well as to the decomposition of electrolytes under high potentials.^43^

Efforts to overcome the low electronic and ionic conductivity of LiCoPO_4_ have included: (1) size reduction and morphology control, decreasing the particle size of LiCoPO_4_ or tailoring its crystal growth orientation along the *a*–*c* plane to decrease the diffusion length of lithium ions in the insertion/extraction process;^44,45^ (2) surface modification (*e.g.* carbon coating), to enhance the electronic conductivity of the composite electrode by forming a conductive network among the LiCoPO_4_ particles;^42,46^ (3) ion doping with cations on either Li or Co sites to enhance the intrinsic electronic/ionic conductivity of LiCoPO_4_ although the mechanism is still in controversy.^29,47^ Among these approaches, the combination of size reduction and conductive agent coating (*e.g.* carbon coating) is regarded as an effective method to enhance the specific capacity and rate capability of LiCoPO_4_ cathode.^48^ Reducing the particle size of LiCoPO_4_ to the nanometer size range can shorten the Li ion transport distance, and thus reduce the time required for Li ion diffusion within the bulk LiCoPO_4_ material. Carbon coating not only improves the surface electrical conductivity of LiCoPO_4_ composite, which alleviates electrode polarization, but also provides effective protection from chemical attack by HF produced *via* electrolyte decomposition at high potentials in LiPF_6_ based electrolytes.^48^ Metal oxides^30,49–52^ and metal nitrides^53–56^ have been combined with other electrode materials to form structured composites with improved conductivity and stability. TiN and RuO_2_ are suitable for this purpose as they have good electrical conductivity, and good chemical and thermal stability.^53,57^

It is important to develop facile, easily scalable and controllable, time and energy saving synthetic routes to produce LiCoPO_4_ with good electrochemical performance.^25^ Various synthesis methods such as hydrothermal/solvothermal syntheses,^42,44^ sol–gel processes^58,59^ and solid-state reactions^60,61^ have been proposed. Hydrothermal/solvothermal synthesis is facile and easily scalable, with mild reaction conditions and advantages of producing nanomaterials with controllable particle sizes and morphologies.^62^ Mixing an organic solvent and water as a co-solvent has been employed in the solvothermal synthesis of LiCoPO_4_.^44,45,62–64^ The solvent mixture can be beneficial for effectively tailoring the particle size of LiCoPO_4_ due to the high viscosity of the organic solvent,^62,63^ and the water component can promote the dissolution of the reagents.^62^ However, optimisation of solvothermal conditions to achieve LiCoPO_4_ cathodes with good specific capacity and cycle performance is still challenging.

Herein, a novel, simple and fast solvothermal approach towards high-performance LiCoPO_4_ at relatively low temperatures (180 °C) using diethylene glycol (DEG) as a co-solvent is presented, followed by thermal treatment under Ar, air, 5%H_2_/N_2_ or NH_3_. Surface modification of LiCoPO_4_ with conductive agents like TiN, RuO_2_ and carbon has been investigated. Unusually in this work the electrochemical performance of samples produced by this method does not require the use of conductive coatings (*e.g.* carbon) to achieve good electrochemical performance.

## Experimental

LiCoPO_4_ was prepared under solvothermal conditions. We previously reported the phase behaviour during charging of a sample made in this way.^65^ LiOH (0.359 g, 0.015 mol, Sigma Aldrich) was dissolved/dispersed in 45 ml deionised water/diethylene glycol (H_2_O/DEG) mixture, then H_3_PO_4_ aqueous solution (0.344 cm^3^, 0.005 mol, 85.3 wt% assay, Fisher Scientific) was added. CoSO_4_·7H_2_O (1.405 g, 0.005 mol, ≥ 99% purity, Sigma Aldrich) was dissolved in 25 ml H_2_O/DEG mixture and added slowly to the LiOH solution with constant stirring, during which time a blue/purple suspension formed. The volume ratio of H_2_O/DEG was set as pure H_2_O, 6 : 1, 3 : 1, 1 : 1, 1 : 3, 1 : 6 and pure DEG. The precursor solution was heated in a Parr 4748 Teflon-lined autoclave (125 cm^3^) at 180 °C for 10 h. The precipitate was then washed with deionized water and ethanol, and dried at 80 °C for 5 h under vacuum. The resulting material was heated at 5 °C min^−1^ to 600 °C and maintained for 3 h under Ar, air, NH_3_ or 5% H_2_/N_2_ to crystallise LiCoPO_4_.

To obtain carbon or RuO_2_ coated LiCoPO_4_ the uncrystallised or pre-fired LiCoPO_4_, (0.3 g, 1.87 mmol) was manually ground in a pestle and mortar with sucrose (C_6_H_12_O_6_, 0.0375 g, 0.11 mmol, Fisher Scientific) or ruthenium(iii) chloride hydrate (RuCl_3_·*x*H_2_O, 0.0246 g, 0.12 mmol, Sigma Aldrich) to obtain a uniform mix that was then heated under Ar as described above. The products were black powders and were ground before further characterisation. TiN modified LiCoPO_4_ powders were prepared using a propylamine cross-linking sol–gel process^53,66–68^ under nitrogen using glove box or Schlenk line conditions. Ti(NMe_2_)_4_ (0.21 cm^3^, 0.9 mmol, prepared from TiCl_4_ and LiNMe_2_) was dissolved in dry THF (7.5 cm^3^, distilled from sodium/benzophenone), and added to 0.5 g dry LiCoPO_4_ powder. ^*n*^PrNH_2_ (0.15 cm^3^, 1.8 mmol, distilled from BaO) was slowly added. The solution gradually changed colour from yellow to red-orange. The suspension was stirred at room temperature for ∼16 h and dried *in vacuo* to form a sticky powder. This was heated under Ar or NH_3_ as described above for LiCoPO_4_ samples.

Powder X-ray diffraction used a Bruker D2 Phaser with CuK_α_ radiation, and data was fitted using the GSAS package.^69^ Scanning electron microscopy (SEM) used a JEOL JSM-6500F (30 kV). Transmission electron microscopy (TEM) used a FEI Tecnai T12 (120 kV). Brunauer–Emmett–Teller (BET) surface area and pore size distribution measurements *via* N_2_ physisorption analysis were carried out with a Micromeritics TriStar II analyser. Electrochemical testing used a Biologics VMP-2 multichannel potentiostat. X-ray photoelectron spectroscopy (XPS) was collected with a two chamber Thermo K-alpha spectrometer with a monochromated Al K-alpha X-ray source (1486.6 eV) in constant analyser energy mode. Sample charging was prevented by use of a dual beam flood gun. X-rays were focused to a 400 μm spot at the sample surface. High resolution core peak spectra were recorded at 50 eV pass energy. Spectra were analysed using Casa XPS software. The binding energy scale was calibrated from the carbon at 285.0 eV. Core peaks were analysed with a nonlinear Shirley-type background.^70^ The peak positions and areas were optimized using a weighted least-square fitting method with 70% Gaussian and 30% Lorentzian line shapes. Several spectral analyses were applied at different positions for each sample to ensure the results were statistically reliable. Electronic and ionic conductivity was determined from the current–voltage measurement and electrochemical impedance spectroscopy on gold-coated sintered LiCoPO_4_ disks (11 mm in diameter and ∼0.5 mm in thickness).^31,71,72^ Current–voltage plots were collected at 20 mV s^−1^ over the range of −0.3 to +0.3 V (or larger voltage ranges) at room temperature. Electrochemical impedance spectroscopies were collected at 500 mV in the frequency range of 0.1 Hz to 200 kHz at room temperature.

Electrodes for use in lithium half cells were prepared by manually mixing the LiCoPO_4_ or TiN/carbon/RuO_2_ coated LiCoPO_4_ powders (75 wt%) with acetylene black (Shawinigan Black, 20 wt%) and polytetrafluoroethylene (6C–N, DuPont, 5 wt%) in a pestle and mortar. The resulting solid paste was hand rolled (Durston Rolling Mill) into a film of ∼90 μm thickness and cut into circular disks with diameter of 11 mm. The pellet was then dried at 120 °C *in vacuo* for 12 h to obtain the cathode with a typical mass of ∼0.022 g. Swagelok cells were assembled in an argon-filled glove box with lithium foil (Rockwood Lithium GmbH) anodes and glass microfiber filter (Whatman, GF/F grade) separators soaked in 8 drops (∼0.4 ml) of 1 mol dm^−3^ LiPF_6_ in ethylene carbonate/ethylmethyl carbonate (EC : EMC = 3 : 7 in weight) electrolyte (BASF, LP57). Galvanostatic testing was carried out at 25 °C at various rates of charge/discharge (*e.g.* 0.1C for a theoretical specific capacity of 167 mA h g^−1^ corresponds to a specific current of 16.7 mA g^−1^) within the voltage range of 3.5–5 V (*vs.* Li/Li^+^).

## Results and discussion

LiCoPO_4_ samples were prepared by a solvothermal method. First, we present a systematic study on the effect of the solvents and heating environment to optimise the solvothermal conditions. Then, LiCoPO_4_ samples were coated with TiN, carbon or RuO_2_ with a variety of processing conditions and thicknesses to determine whether the expected conductivity enhancement and increased surface stability improved the electrochemical behaviour of the materials.

### Effect of solvent on LiCoPO_4_ morphology in solvothermal synthesis

Uncoated LiCoPO_4_ samples were produced using H_2_O/DEG solvent mixtures with various volume ratios, followed by firing at 600 °C in an Ar environment, to determine the effect of solvents on their morphologies. The volume ratio of H_2_O/DEG in solvothermal synthesis was set as pure H_2_O, 6 : 1, 3 : 1, 1 : 1, 1 : 3, 1 : 6 and pure DEG, which corresponds to samples defined as LCP-H_2_O(Ar), LCP-6 : 1(Ar), LCP-3 : 1(Ar), LCP-1 : 1(Ar), LCP-1 : 3(Ar), LCP-1 : 6(Ar), LCP-DEG(Ar), respectively. The heating temperature affects purity, crystallite/particle size distribution and specific capacity of LiCoPO_4_.^53^ Most successful previous studies produce LiCoPO_4_ samples at 550–700 °C,^45,73–75^ and in this study samples were fired at 600 °C.

**Fig. 1:**
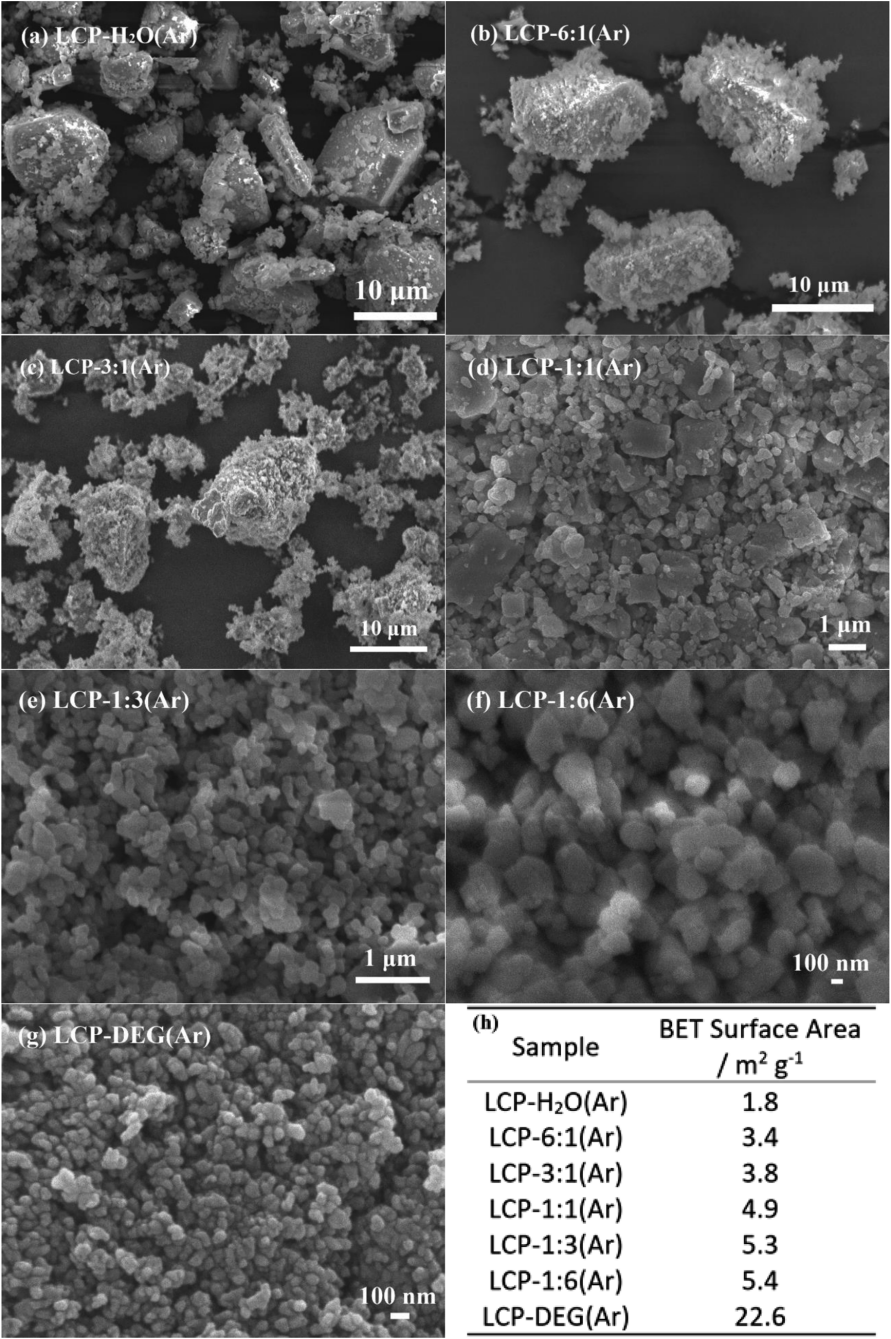
(a–g) SEM images of LiCoPO_4_ samples synthesised by the solvothermal method, using H_2_O/DEG solvent mixture with various volume ratios, followed by firing at 600 °C in Ar. (h) BET surface area of LiCoPO_4_ samples.

The SEM images (Fig. 1) show the morphologies of LiCoPO_4_ samples obtained using various ratios of H_2_O/DEG. The particle size of LiCoPO_4_ decreased from ∼10 μm to ∼80 nm with increasing DEG content (Fig. 1a–g), and its BET surface area increased from 1.8 to 22.6 m^2^ g^−1^ (Fig. 1h). As the ratio of H_2_O/DEG decreases to less than 1 : 3, the particle size distribution of LiCoPO_4_ becomes homogeneous (Fig. 1f and g). LiCoPO_4_ particles readily grow to large sizes in hydrothermal (pure water) synthesis.^42,76–78^ The pore size distribution of LiCoPO_4_ samples obtained using various ratios of H_2_O/DEG were investigated *via* N_2_ physisorption analysis (ESI, Fig. S1†). The isotherms of LiCoPO_4_ samples belong to the type-II, which is reflective of nonporous or macroporous structure. The density functional theory (DFT) pore size distributions calculated from the adsorption curves reveal that the main pore sizes of LiCoPO_4_ samples are 4–20 nm. These mesopores are created by the interfaces between nonporous LiCoPO_4_ particles. The control of particle sizes in solvent mixtures has been attributed to the increased viscosity of the solvent mixture when increasing DEG concentration, which can reduce mass transport to growing crystallite surfaces, thus results in decreasing LiCoPO_4_ particle size.^63,79^ Also, the solubility of the precursors decreases as the solvent mix becomes less polar, which increases the nucleation rate during the solvothermal process.^45^ For a given amount of precursor, more nuclei means less matter for each nucleus.^45,80^ Therefore, larger nucleation rate in solvothermal process could result in smaller LiCoPO_4_ particle size.

Our previous review on LiCoPO_4_ inferred that good rate capability is more likely to be achieved by LiCoPO_4_ with particle size less than 200 nm.^25^ For example, Wei *et al.* synthesized carbon coated LiCoPO_4_ with particle size of 150 nm *via* a microwave heating method. This nanostructured LiCoPO_4_ provides a specific capacity of 144 mA h g^−1^ at 0.1C, with reasonable rate capability of 116, 90 and 71 mA h g^−1^ at 5, 10 and 20C, respectively.^81^ In this work, sample LCP-1 : 6(Ar) and LCP-DEG(Ar) showed homogeneous particle size distribution with nanoparticle of less than 200 nm. This small particle size can reduce the length of Li-ion migration paths, and facilitate easier Li-ion transfer in LiCoPO_4_ crystals, thus enhancing the rate performance of LIBs.^45,79,82^ However, nanosized LiCoPO_4_ particles with high surface area can enlarge the electrode/electrolyte interface area, which leads to undesirable electrode/electrolyte by-reactions, thus resulting in a poor cycle stability.^20^ Hence, sample LCP-1 : 6(Ar) with particle size of ∼150 nm and a relatively small surface area of 5.4 m^2^ g^−1^ (compared to LCP-DEG(Ar) with surface area of 22.6 m^2^ g^−1^) was chosen for the following studies.

### Effect of heating environment on bare LiCoPO_4_

**Scheme 1:**
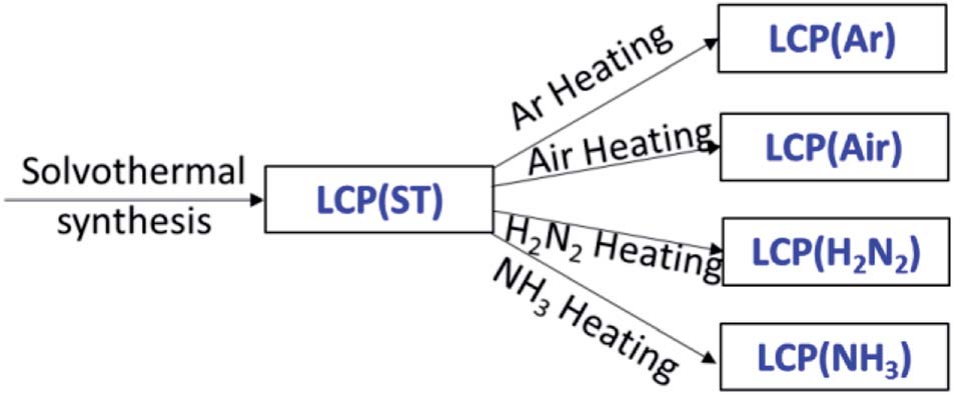
Solvothermal synthesis to prepare LiCoPO_4_, using 1 : 6 (v/v) H_2_O/DEG co-solvent, followed by firing at 600 °C in Ar, air, 5% H_2_/N_2_ or NH_3_.

Ar or air are typical heating environments in thermal treatment to crystallise LiCoPO_4_, but the intrinsic role and effects of various heating gases on LiCoPO_4_ has still not been fully ascertained and remains controversial.^25^ NH_3_ and 5% H_2_/N_2_ are typical heating gases to coat TiN and carbon onto electrode materials.^25,48,53^ Thus, it is important to evaluate whether heating in NH_3_ or 5% H_2_/N_2_ caused a deterioration in the LiCoPO_4_ properties. In this section, uncoated LiCoPO_4_ samples were produced by using the 1 : 6 (v/v) H_2_O/DEG co-solvent optimised above, and fired at 600 °C in Ar, air, 5% H_2_/N_2_ or NH_3_ to determine the effect of heating environment on their behaviour. Scheme 1 shows the labels used for different samples.

**Fig. 2:**
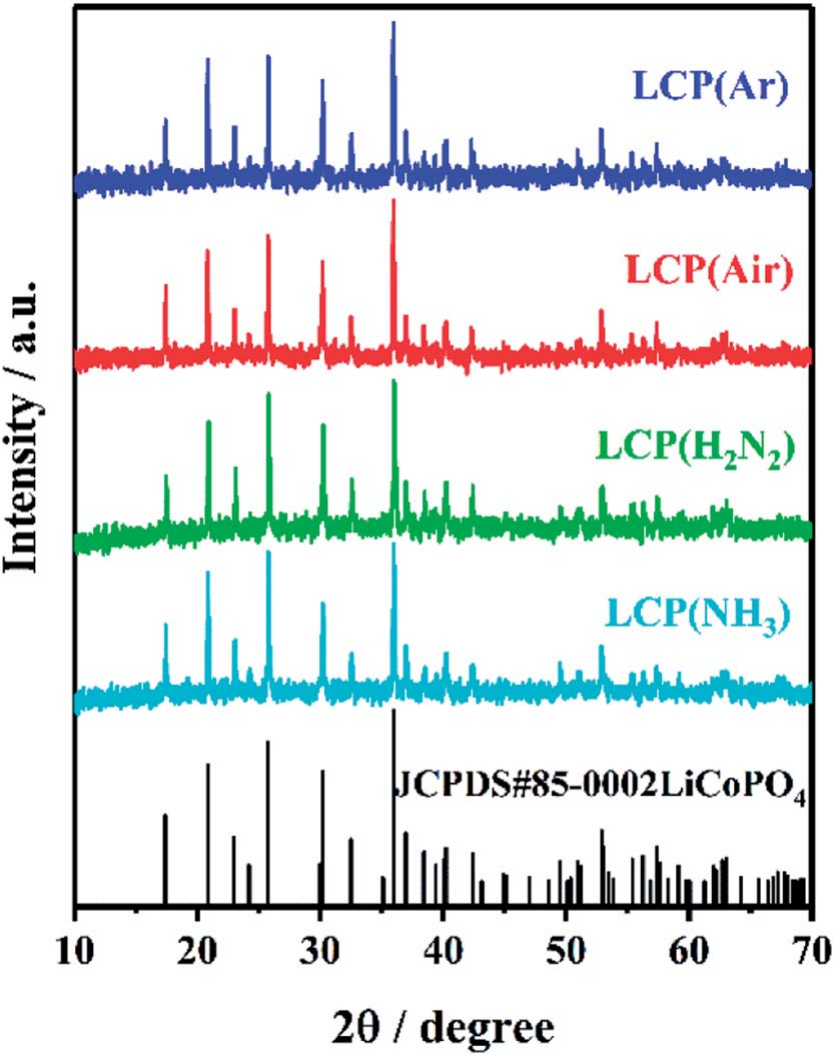
XRD patterns of LiCoPO_4_ samples heated under Ar, air, 5% H_2_/N_2_ and NH_3_, respectively, at 600 °C (labels explained in Scheme 1). The black stick pattern denotes the literature positions and intensities of LiCoPO_4_ reflections.^85^

**Fig. 3:**
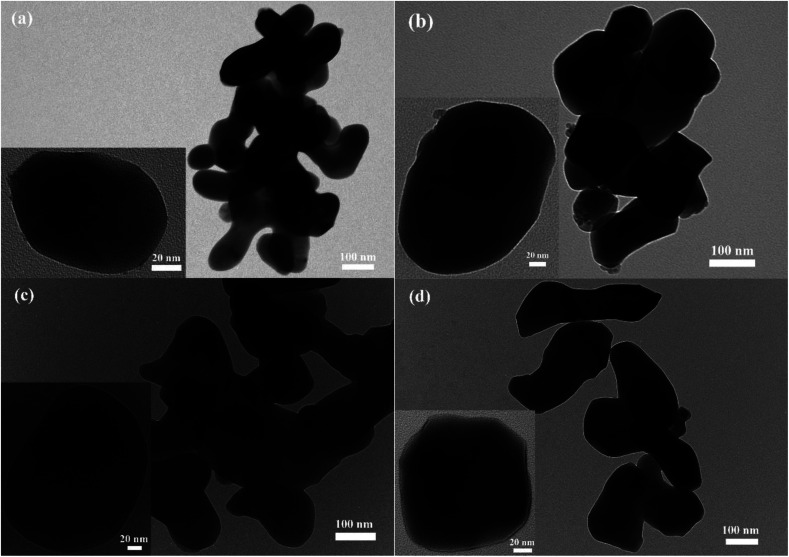
TEM images of (a) LCP(Ar), (b) LCP(air), (c) LCP(H_2_N_2_) and (d) LCP(NH_3_) (scale bar = 100 nm). (Inset) magnified TEM images of single LiCoPO_4_ particle (scale bar = 20 nm). Sample labels are explained in Scheme 1.

The X-ray diffraction peaks of the resulting LiCoPO_4_ samples (Fig. 2) were consistent with the standard olivine LiCoPO_4_ (JCPDS card no. 85–0002, space group *Pnma*) as expected. Table S1† shows the crystallographic data of LiCoPO_4_ samples. The Rietveld fits^83^ to this XRD data (ESI, Fig. S2†) resulted in similar lattice parameters (ESI, Table S1†) to those in the literature for LiCoPO_4_ indicating that the heating environment did not affect the crystal structure of LiCoPO_4_.^84^ The Lorentzian peak broadening in the Rietveld fit indicated average LiCoPO_4_ crystallite sizes of 119–132 nm. These were consistent with TEM (Fig. 3) and SEM (ESI, Fig. S3†) images of LiCoPO_4_ fired in Ar, air, 5%H_2_/N_2_ and NH_3_, which showed particle sizes of ∼150 nm. No hydrogen and nitrogen (<0.1 wt%) are detectable according to the microanalysis results (ESI, Table S1†) with a negligible amount of carbon (<0.5 wt%) in the LiCoPO_4_ samples.

**Fig. 4:**
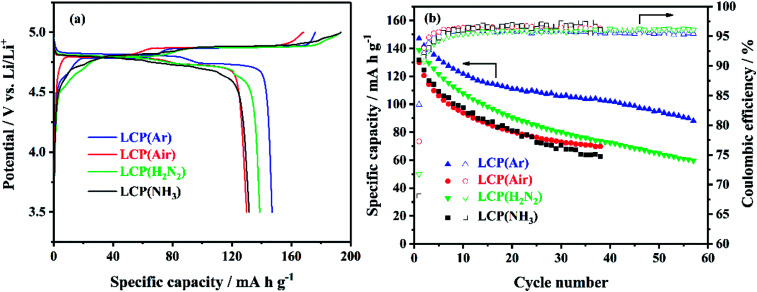
(a) The initial cycle voltage profile *vs.* specific capacity and (b) specific capacity and coulombic efficiency *vs.* cycle number of LiCoPO_4_/Li half cells under galvanostatic cycling between 3.5 and 5 V at 0.1C (sample labels explained in Scheme 1).

The electrochemical performance of LiCoPO_4_ samples was assessed by galvanostatic cycling of Li half cells. The initial charge/discharge curves and the variations in discharge capacity and coulombic efficiency 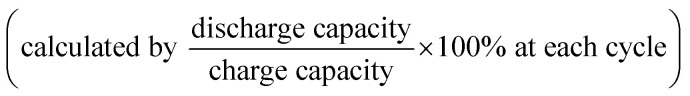 over the first 40 cycles of LiCoPO_4_ fired in Ar, air, 5% H_2_/N_2_ and NH_3_ are shown in Fig. 4. LCP(Ar), LCP(air), LCP(H_2_N_2_) and LCP(NH_3_) had initial discharge capacities of 147, 130, 139 and 132 mA h g^−1^, respectively. The capacity of LCP(Ar) decayed gradually with continuous cycling, retaining 102 mA h g^−1^ after 40 cycles, and 88 mA h g^−1^ after 57 cycles. The low coulombic efficiency values in the first cycle for these samples are caused by the decomposition of the electrolyte during charge at high potentials.^62,86^ The coulombic efficiency of LCP(Ar), which improved upon cycling, was 92% in the second cycle and maintains values higher than 95% after five cycles. LiCoPO_4_ fired in air or in reducing gases had lower initial discharge capacities and lost capacity more rapidly on cycling. A comparison of relevant articles using a hydrothermal/solvothermal methodology in the synthesis of LiCoPO_4_ olivine phosphate cathodes is presented in Table 1. The obtained specific capacity and cycle stability of uncoated LCP(Ar) in our case is comparable or higher than most previous studies, even though in most of these reports LiCoPO_4_ has been optimised with conductive coatings (*e.g.* carbon). Overall the results suggested that Ar firing was the most effective heat treatment to apply for the crystallisation of LiCoPO_4_, but since air firing is also common in this system,^75,87–89^ both samples as well as uncrystallised LiCoPO_4_ were carried forward to test the surface modification of LiCoPO_4_ with TiN, RuO_2_ and carbon.

**Table 1:** Morphologies and electrochemical behaviours of LiCoPO_4_ samples synthesised under hydrothermal/solvothermal conditions (shown in chronological order with the most recent study first)

Morphology, particle size	Rate performance, mA h g^−1^	Cycle stability	Ref.
Nanoparticles, 150 nm	147 (0.1C)	102 mA h g^−1^ at 0.1C after 40 cycles	This work
Irregular nanoparticle	160 (0.1C), 138 (1C), 120 (2C), 88 (5C)	138 mA h g^−1^ at 0.1C after 100 cycles	73
Hexagonal platelets, 200 × 100 × 50 nm to 1.2 × 1.2 × 0.5 μm	136 (0.1C), 125 (0.2C), 115 (0.5C), 105 (1C), 95 (2C)	108 mA h g^−1^ at 0.5C after 15 cycles	63
Square, rhombic and hexagonal platelets, 600–800 × 400–600 × 100–150 nm to 9 × 7 × 3 μm	141 (0.1C), 135 (0.2C), 130 (0.5C), 123 (1C), 112 (2C)	125 mA h g^−1^ at 0.5C after 15 cycles	44
Spherical or oblong spheroid, 50–250 nm	145 (0.1C)	74 mA h g^−1^ at 0.1C after 20 cycles	90
Irregular particles, 390 nm to 2.8 μm	135 (0.1C), 132 (0.5C), 125 (1C), 117 (2C), 101 (5C)	70 mA h g^−1^ 0.1C after 30 cycles	42
Irregular particles, 200 nm to 1 μm	155 (0.1C), 129 (1C), 98 (5C), 70 (10C), 51 (20C)	141 mA h g^−1^ at 0.1C after 80 cycles	74
Particles, 100–500 nm	97 (0.1C)	82 mA h g^−1^ at 0.1C after 20 cycles	85
Particles, 500 nm to 10 μm	124 (0.1C), 111 (0.5C), 100 (1C), 85 (2C), 51 (5C)	103 mA h g^−1^ at 0.1C after 100 cycles	45
Hexagonal platelets, 400–600 × 700–800 × 100–220 nm	137 (0.1C), 114 (0.5C), 97 (2C)	78 mA h g^−1^ at 0.5C after 100 cycles	62
Hexagonal platelets, thickness < 200 nm	120 (0.1C), 85 (0.5C), 75 (1C)	90 mA h g^−1^ at 0.1C after 10 cycles	64
Flower-like, 5–10 μm (compose of plate-like, 1–2 μm × 200 nm)	107 (0.05C), 60 (2C)	30 mA h g^−1^ at 0.05C after 20 cycles	78
Hexagonal/octagonal platelet, thickness of 50–100 nm	95 (0.1C), 76 (0.5C)	75 mA h g^−1^ at 0.1C after 10 cycles	91 and 92
Nanoparticles agglomeration, 2–3 μm	105 (0.2C)	95 mA h g^−1^ at 0.2C after 30 cycles	93
Hedgehog-like, 5–8 μm (compose of nanorods, 40 nm × 1 μm)	136 (0.1C), 85 (5C)	124 mA h g^−1^ at 0.1C after 50 cycles	94
Rod, 300–700 nm × 5 μm	65 (0.1C)	50 mA h g^−1^ at 0.1C after 10 cycles	77
Cubes, 1.2–1.5 μm × 250 nm	52 (0.1C)	15 mA h g^−1^ at 0.1C after 25 cycles	76

### Synthesis, microstructure and electrochemistry of LiCoPO_4_ modified with TiN, RuO_2_ or C

**Scheme 2:**
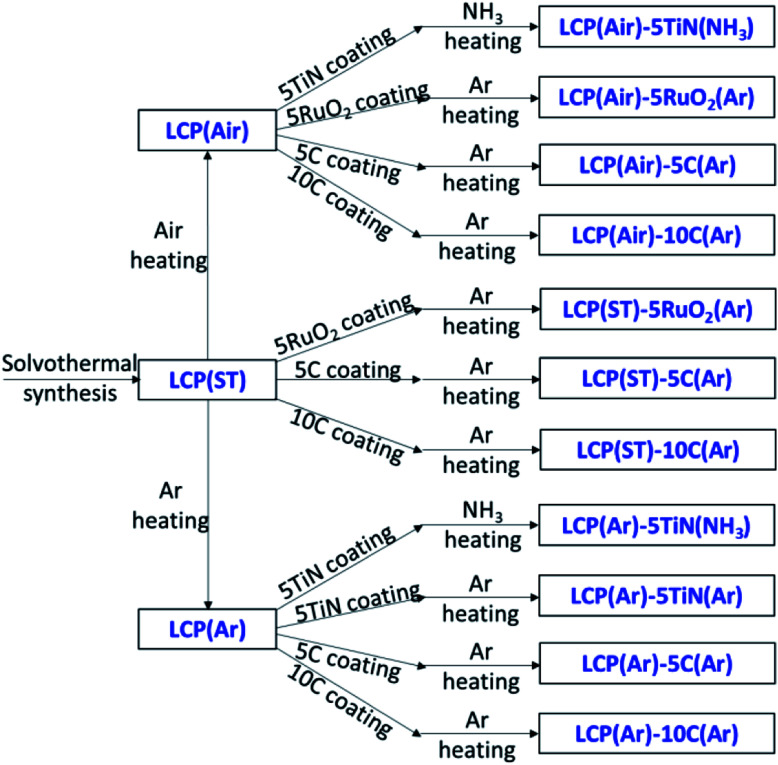
Preparation conditions and sample labels for TiN, RuO_2_ or C coated LiCoPO_4_ materials.

Three kinds of LiCoPO_4_ were chosen for coating, the uncrystallised LiCoPO_4_ directly after solvothermal synthesis, with the advantage of a single heating step, and the LiCoPO_4_ already crystallised in Ar or air (Scheme 2). RuO_2_ and C coatings were prepared by manually grinding the precursors (RuCl_3_·xH_2_O or sucrose) together with LiCoPO_4_, then firing under Ar.^45,57^ This solid-state process proved to be an easy and effective method to achieve carbon coatings on LiCoPO_4_.^25,45^ TiN coating used a propylamine-crosslinked sol–gel method, then firing under Ar or NH_3_. This sol–gel process has been shown to be effective to achieve TiN coatings onto LIB cathode materials according to our previous research.^53^Scheme 2 summarises these approaches.

**Fig. 5:**
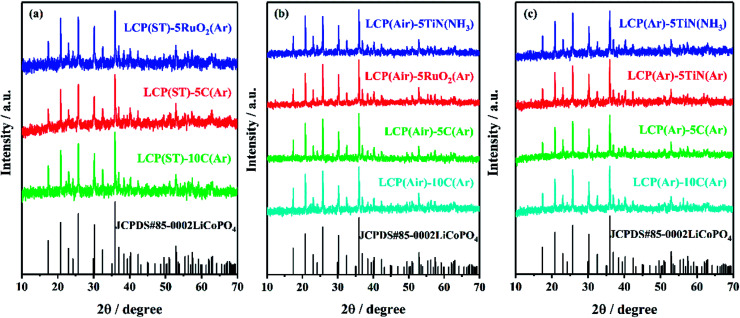
XRD patterns of uncrystallised LiCoPO_4_ directly after solvothermal synthesis (left), and LiCoPO_4_ heated under air (centre) or Ar (right) at 600 °C, then modified with TiN, RuO_2_ and carbon, respectively (labels explained in Scheme 2). The black stick pattern denotes the literature positions and intensities of LiCoPO_4_ reflections.^85^

All the X-ray diffraction peaks of the resulting TiN, RuO_2_ and C coated LiCoPO_4_ samples (Fig. 5) can be indexed to the standard olivine LiCoPO_4_ structure. The characteristic peaks of TiN and RuO_2_ were not detectable in coated LiCoPO_4_ composites due to their low concentrations. Carbon coatings on battery materials are typically amorphous when heating at around 600 °C,^48,61,81^ and also were not visible in the diffraction data. Fig. S4–S6† show the Rietveld fits to the XRD data, which yielded typical LiCoPO_4_ lattice parameters (ESI, Tables S2–S4†),^84^ suggesting that the coating processes did not affect the crystal structure of LiCoPO_4_.

**Fig. 6:**
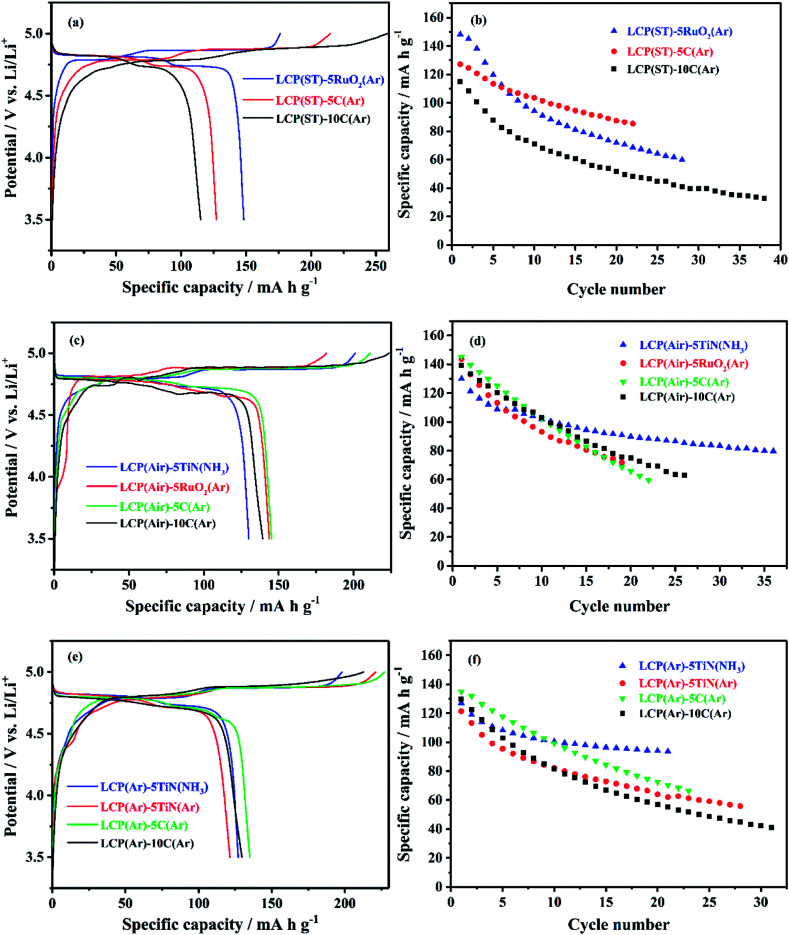
(a), (c) and (e) The initial cycle voltage profile *vs.* specific capacity, and (b), (d) and (f) specific capacity *vs.* cycle number of TiN, RuO_2_ or C coated LiCoPO_4_ samples made into Li half cells, under galvanostatic cycling between 3.5 and 5 V at 0.1C (sample labels explained in Scheme 2, with the percentage of TiN, RuO_2_ or C in the composite written after the hyphen).

Fig. 6 shows the initial charge/discharge curves at 0.1C and the cycle stability of electrodes produced from the coated materials. Carbon is the most commonly used battery material coating, but RuO_2_ has been used to coat electroactive materials to offer a high electronic conductivity and quick Li permeation.^95–98^ Due to its good electrical conductivity, chemical stability and thermal stability, TiN has been combined with other electrode materials to form structured composites with improved conductivity and stability.^53,55,56^

RuO_2_ coating of the unfired LiCoPO_4_ (Fig. 6a and b) resulted in a higher initial discharge capacity of 148 mA h g^−1^ as expected due to the utility of RuO_2_ in generating very effective mixed conducting heterogeneous electrodes.^57^ However, its capacity drops quickly in subsequent cycles. The carbon coated samples had lower capacities than their uncoated counterparts, and the drop in capacity when C content was increased from 5% to 10% suggests that the thicker carbon coating hindered lithium diffusion.

Air fired LiCoPO_4_ samples coated with TiN, RuO_2_, 5 wt% C or 10 wt% C (Fig. 6c and d) had initial discharge capacities of 130, 144, 145 and 139 mA h g^−1^, respectively. The TiN coated sample retained a fairly large fraction of the initial capacity during continuous cycling.^53^ However, the cycle stability was quite similar to the uncoated LCP(air) (Fig. 4), so the coatings did not significantly improve the electrochemical performance of LiCoPO_4_. Notably cycle stability was less good with RuO_2_ or C coatings than with uncoated material.

A similar position was observed with the Ar-fired LiCoPO_4_ (Fig. 6e and f). The capacities of the C or TiN coated samples dropped to around 100 mA h g^−1^ over 10 cycles, a poorer cycle stability than that of the uncoated LCP(Ar), which retained 102 mA h g^−1^ after 40 cycles (Fig. 4). The coatings did not deliver the expected improvement in electrochemical performance of LiCoPO_4_. However, the purpose of the conductive agent coating was to create a conductive network among the LiCoPO_4_ particles to improve the conductivity of the composites. These results show that, using these optimised solvothermal conditions, the conductivity of the bare LCP(Ar) sample is good enough to provide competitive specific capacity and cycle stability.

### Further investigation of LiCoPO_4_ fired in Ar and air

**Fig. 7:**
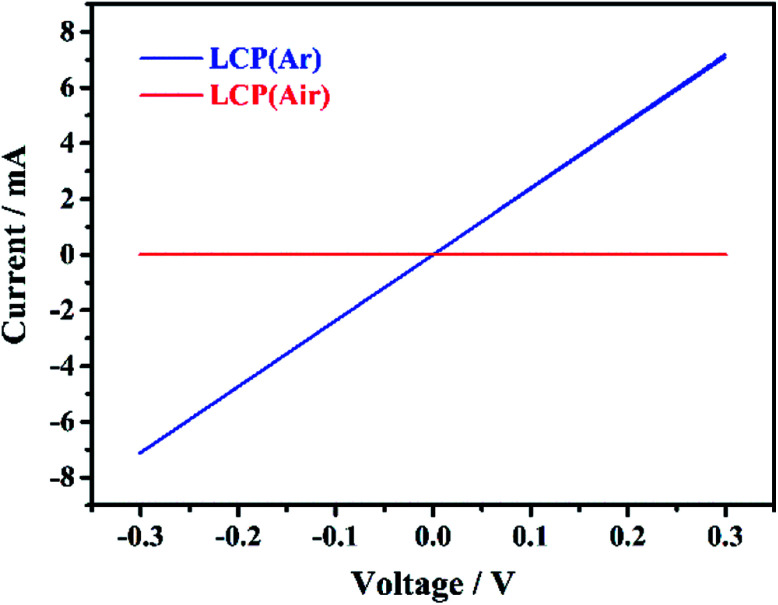
Current–voltage plots (3 cycles each) for LiCoPO_4_ samples fired in Ar and air, respectively, cycling at scanning rate of 20 mV s^−1^, showing the ohmic behaviour of the samples (labels explained in Scheme 1).

The electronic and ionic conductivity of LiCoPO_4_ powders fired in Ar and air was evaluated with current–voltage measurements and electrochemical impedance spectroscopy. LiCoPO_4_ samples were pressed, sintered and gold-coated to produce self-standing LiCoPO_4_ disks, which were dry contacted with two silver paste electrodes. The linear current–voltage plots (Fig. 7) showed that the LCP(Ar) pellet behaves as a resistor and the current–voltage relationship is given by Ohm's law: *V* = *IR*. Hence, the resistance of the LiCoPO_4_ samples can be estimated as being equal to the inverse of the slope of the current–voltage plot. The conductivity of the samples is given by 
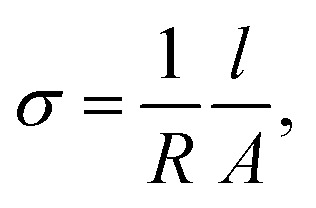
 where *σ* is the conductivity, *l* is the thickness of the LiCoPO_4_ pellets (0.48 mm for LCP(Ar) and 0.64 mm for LCP(air) sample), *A* is the area of the LiCoPO_4_ pellets (95 mm^2^), and *R* is the resistance (42.2 Ω for LCP(Ar) and 1.7 × 10^7^ Ω for LCP(air) sample). The conductivities of LCP(Ar) and LCP(air) are calculated to be ∼10^−3^ S cm^−1^ and ∼10^−9^ S cm^−1^, respectively. Current–voltage plots with larger voltage ranges are shown in Fig. S7,† and they are in agreement with those in Fig. 7.

These conductivity results can be confirmed by electrochemical impedance spectroscopy measurements of the gold-coated pressed LiCoPO_4_ pellets, as presented in Fig. S8.† The impedance of the LCP(Ar) sample shows purely resistor behaviour (ESI, Fig. S8a†). This is in agreement with the fact that this sample has reasonably high electronic conductivity of ∼10^−3^ S cm^−1^, estimated from the value of the resistance and taking into account the dimension of the pellet. On the other hand, the LCP(air) sample shows much higher values of impedance (ESI, Fig. S8b†). This is ascribed to the fact that this sample has much higher electronic resistance, thus it behaves as a resistor coupled to a capacitor (or a constant phase element) in parallel. In addition, the surface of the pellet cannot be polished prior to gold coating (due to the fragility of the pellet), thus the LiCoPO_4_–gold interphase behaves as a Warburg element, rather than a capacitor or a constant phase element. By fitting the data to the equivalent circuit shown in Fig. S8b,† the electronic conductivity of the LCP(air) sample is estimated to ∼10^−9^ S cm^−1^. This is in agreement with the estimation of the total conductivity of the samples by using current–voltage measurements, and the dramatic difference in conductivity between these two samples explains the fact that the sample fired in Ar showed better specific capacity and cycling performance.

Wolfenstine *et al.* investigated the effect of added carbon on the electronic conductivity and specific capacity of LiCoPO_4_, and found that the added carbon was partly consumed to reduce the LiCoPO_4_ surface layers to Co_2_P during heating under Ar atmosphere.^33,34^ The formation of highly conductive (∼10^−1^ S cm^−1^) Co_2_P phase in LiCoPO_4_ cathode led to improved electrochemical performance. As the amount of the Co_2_P phase increased to 4 wt%, the electronic conductivity increased to ∼10^−4^ S cm^−1^ with a maximum discharge capacity of ∼120 mA h g^−1^ obtained. However, for LiCoPO_4_ cathodes with higher concentrations of Co_2_P, the capacities dropped rapidly due to the electrochemically inert Co_2_P phase, which improves the electronic conductivity but tends to hinder the Li^+^ insertion/extraction. Similar phenomena were also observed by Xu^99^ and Indris *et al.*^88^ Ma *et al.* demonstrated that the presence of Co_2_P can accelerate the electrolyte decomposition at high voltage in the charge process for LiCoPO_4_ due to the catalytic property of Co_2_P.^100^ Dimesso *et al.* suggested that the formation of Co_2_P occurs due to reduction reactions at the grain boundaries of the LiCoPO_4_ crystalline phase during annealing at high temperatures.^101–107^ Brutti *et al.* synthesized LiCoPO_4_*via* a solvothermal synthesis followed by heating under Ar atmosphere. It was found that the heating promotes Co_2_P precipitation on the LiCoPO_4_ particles surface together with loss of organic by-products formed in the solvothermal synthesis.^108^ Nallathamby *et al.* confirmed that the presence of Co_2_P as a second phase enhanced the conductivity and electrochemical performance of LiCoPO_4_. It was found that the Co_2_P is achievable only in an inert atmosphere. The LiCoPO_4_ cathode showed a discharge capacity of 123 mA h g^−1^ at 0.1C with capacity retention of 89% after 30 cycles, and rate capability of 81 mA h g^−1^ at 5C.^61^

**Fig. 8:**
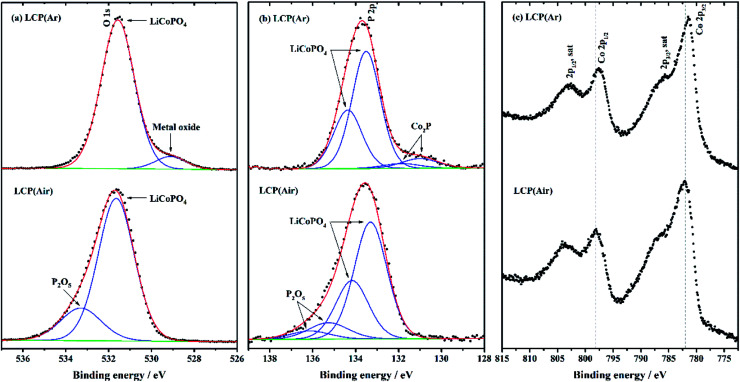
P 2p, O 1s and Co 2p XPS spectra of LiCoPO_4_ samples fired in Ar and air, respectively (labels explained in Scheme 1). The data points and enveloped fitting plot are overlaid in black dots and a red line, respectively. The fitting peaks and background are shown in blue and green, respectively.

Based on the discussion above, the better conductivity of LiCoPO_4_ heated in Ar was considered likely to be due to the presence of Co_2_P on the surface on LiCoPO_4_. X-ray photoelectron spectroscopy (XPS) of LiCoPO_4_ samples fired under Ar and air is shown in Fig. 8. These two samples had similar Li 1s and C 1s spectra (ESI, Fig. S9†). Particularly, for the LiCoPO_4_ fired under Ar, the Li 1s signal located at 55.7 eV is well in accordance with the value reported for LiCoPO_4_.^61^ The C 1s spectrum consists of three peaks, with the main component at 285.0 eV corresponding to C–C, and the other two peaks observed at 287.1 eV and 289.0 eV attributed to C–O and O=C–O environments of carbon.^109^Fig. 8 shows clear differences between the chemical environments present in the P 2p and O 1s spectra of these samples. For the LiCoPO_4_ fired under Ar, the O 1s spectrum with a binding energy of 531.6 eV is in agreement with the air-fired sample and with the (PO_4_)^3−^ environment in LiCoPO_4_,^61^ but an additional weak peak at 529.1 eV demonstrates the presence of a small amount of metal oxide (*e.g.* Li_2_O with binding energy of 528.6 eV for O 1s spectrum).^110–112^ The P 2p spectrum (2p_3/2_ and 2p_1/2_ doublet) shows the main component at 133.5–134.4 eV in accordance with LiCoPO_4_,^91^ and a doublet at lower binding energy (130.9–131.8 eV) that corresponds to Co_2_P.^113^ A small shift in binding energy of P 2p in Co_2_P (expected at around 129 eV) is likely to be due to a differential charging effect resulting from the different electrical conductivities at the surfaces of LiCoPO_4_ and Co_2_P.^113–115^ Co_2_P formation in Ar fired LiCoPO_4_ is attributed to the carbon-containing organic solvent (DEG) chosen for the synthesis, which can decompose at high temperature and the resulting carbon can cause carbothermal reduction to reduce the LiCoPO_4_ surface layers to Co_2_P during heating under inert atmosphere.^33,34,61,101,105,108^ This process also explains the relatively low carbon content measured in these samples by microanalysis. The Co 2p spectra are not fitted due to the complexity of the 2p spectra resulting from peak asymmetries, complex doublet splitting, shake-up and plasmon loss structure, and uncertain, overlapping binding energies.^116^ For the LiCoPO_4_ fired under Ar, the 2p_3/2_ and 2p_1/2_ doublet in the Co 2p spectrum has binding energy values of 781.5 and 797.6 eV, respectively. In LCP(air) these peaks are observed at 782.1 eV and 798.1 eV (2p_3/2_ and 2p_1/2_). The shift to lower binding energy can be attributed to the presence of Co_2_P in LCP(Ar).^113^ The difference of binding energy between Co 2p_3/2_ and its satellite peak is in agreement with the Co^2+^ environment in LiCoPO_4_.^91,116^ For the LiCoPO_4_ fired under air, a new chemical environment corresponding to P_2_O_5_ is identified with additional peaks in the P 2p (135.3–136.1 eV) and O 1s (533.3 eV) spectra.^117,118^

The best specific capacity (147 mA h g^−1^) and cycling performance of LiCoPO_4_ shown in Fig. 4, achieved by heating in Ar, can be attributed the good electronic conductivity (∼10^−3^ S cm^−1^) of LiCoPO_4_ due to the presence of Co_2_P after firing under Ar. Also, the nanosized LiCoPO_4_ obtained from DEG promoted solvothermal synthesis provides short Li-ion migration paths, and facilitates easier Li-ion transfer within the material. The LiCoPO_4_ fired in air showed relatively low initial specific capacity of 130 mA h g^−1^. This could be attributed to the poor electric conductivity of ∼10^−9^ S cm^−1^ (Fig. 7 and S8†) as there is no evidence of the presence of Co_2_P in this sample.

## Conclusions

A facile solvothermal synthesis to prepare olivine-structured LiCoPO_4_ for high-voltage cathodes in LIBs has been developed, using various ratios of water/diethylene glycol as solvent, followed by thermal treatment under Ar, air, 5% H_2_ + N_2_ or NH_3_. The diethylene glycol plays an important role in tailoring the particle size of LiCoPO_4_. It is found that using a ratio of water/diethylene glycol of 1 : 6 (v/v), LiCoPO_4_ is obtained with a homogenous particle size of ∼150 nm. The LiCoPO_4_ prepared after heating in Ar exhibits high initial discharge capacity of 147 mA h g^−1^ at 0.1C with capacity retention of 70% after 40 cycles. This is attributed to the enhanced electronic conductivity of LiCoPO_4_ due to the presence of Co_2_P after firing under Ar. The specific capacity and cycle stability of carbon, TiN and RuO_2_ coated LiCoPO_4_ were also examined, but did not improve the performance of the material. Hence, under our solvothermal synthesis conditions, LiCoPO_4_ with good discharge capacity and cycle stability, without need for separate conductivity coatings, were produced.

## Conflicts of interest

There are no conflicts of interest to declare.

## Supplementary Material

RA-009-C8RA08785G-s001
