# Supplementary material for: Solvothermal water-diethylene glycol synthesis of LiCoPO4 and effects of surface treatments on lithium battery performance
Source: RSC Adv. 2019 Jan 4;9(2):740–52. doi: 10.1039/c8ra08785g (PMC9059495; doi:10.1039/c8ra08785g)
Supplement: RA-009-C8RA08785G-s001 [file RA-009-C8RA08785G-s001.pdf]

Electronic Supplementary Material (ESI) for RSC Advances.
This journal is © The Royal Society of Chemistry 2018

Supplementary information: Solvothermal water-diethylene glycol synthesis of $LiCoPO_4$ and effects of surface treatments on lithium battery performance

Min Zhang,[a] Nuria Garcia-Araez,[a] Andrew L. Hector,*[a] John R. Owen,[a] Robert G. Palgrave,[b] Michael G. Palmer[a] and Samantha Soulé[a]

[a] School of Chemistry, University of Southampton, Highfield, Southampton SO17 1BJ, UK

[b] Department of Chemistry, University College London, 20 Gordon Street, London WC1H 0AJ, UK

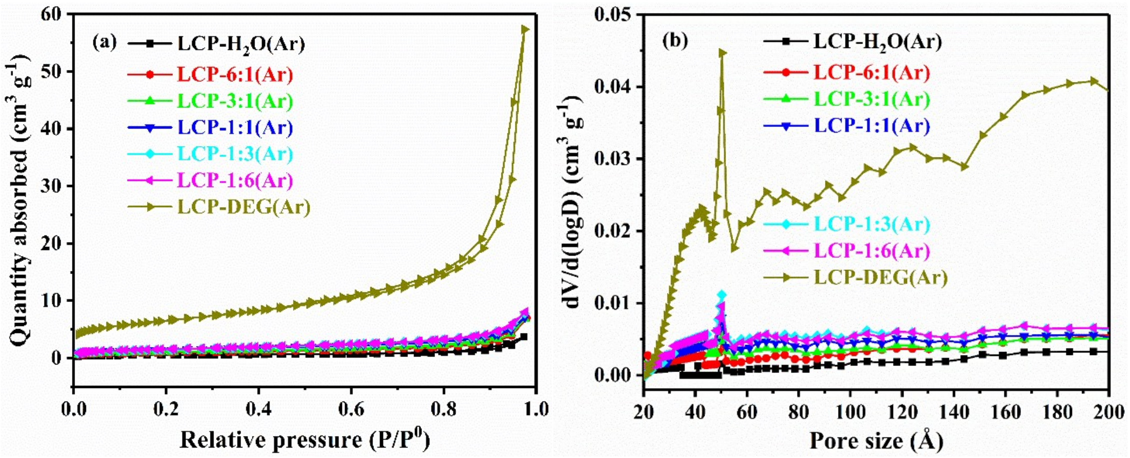

Figure S1 (a) N_2 physisorption isotherms and (b) pore size distribution (DFT method) curves of $LiCoPO_4$ samples obtained using various ratios of H_2O/DEG.

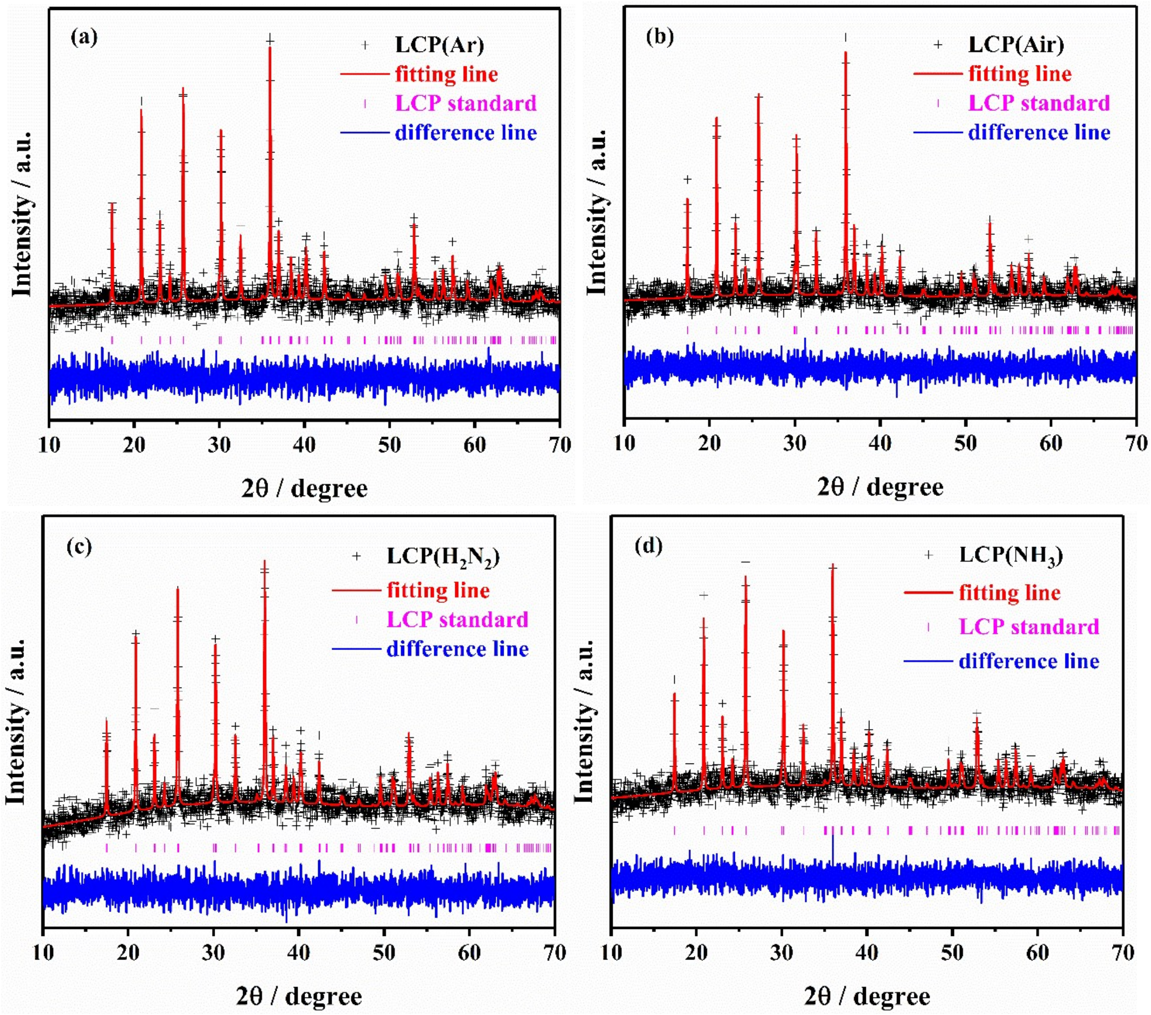

Figure S2 Rietveld fits to the XRD patterns of (a) LCP(Ar) (R_{wp} = 2.81% and R_p = 2.25%), (b) LCP(Air) (R_{wp} = 2.78% and R_p = 2.22%), (c) LCP(H_2N_2) (R_{wp} = 2.72% and R_p = 2.15%) and (d) LCP(NH_3) (R_{wp} = 2.65% and R_p = 2.12%) samples (sample labels explained in Scheme 1). The data points and Rietveld fits are overlaid in black crosses and red lines, respectively. The difference plots are shown in blue. The pink tick marks represent the allowed reflection positions for $LiCoPO_4$ with space group Pnma.

Table S1 Lattice parameters and crystallite sizes obtained from the Rietveld fits to the XRD patterns, and combustion analysis (C, H, N) results of the $LiCoPO_4$ samples (labels explained in Scheme 1).

Sample	a / Å	b / Å	c / Å	Crystallite size / nm	% C	% H	% N
LCP(Ar)	10.2076(7)	5.9248(3)	4.7015(3)	132(5)	0.39	<0.10	<0.10
LCP(Air)	10.2060(6)	5.9248(3)	4.7013(3)	120(3)	0.38	<0.10	<0.10
LCP(H_2N_2)	10.2088(7)	5.9277(4)	4.7038(3)	119(4)	0.49	<0.10	<0.10
LCP(NH_3)	10.2067(7)	5.9257(4)	4.7004(3)	129(5)	0.43	<0.10	<0.10

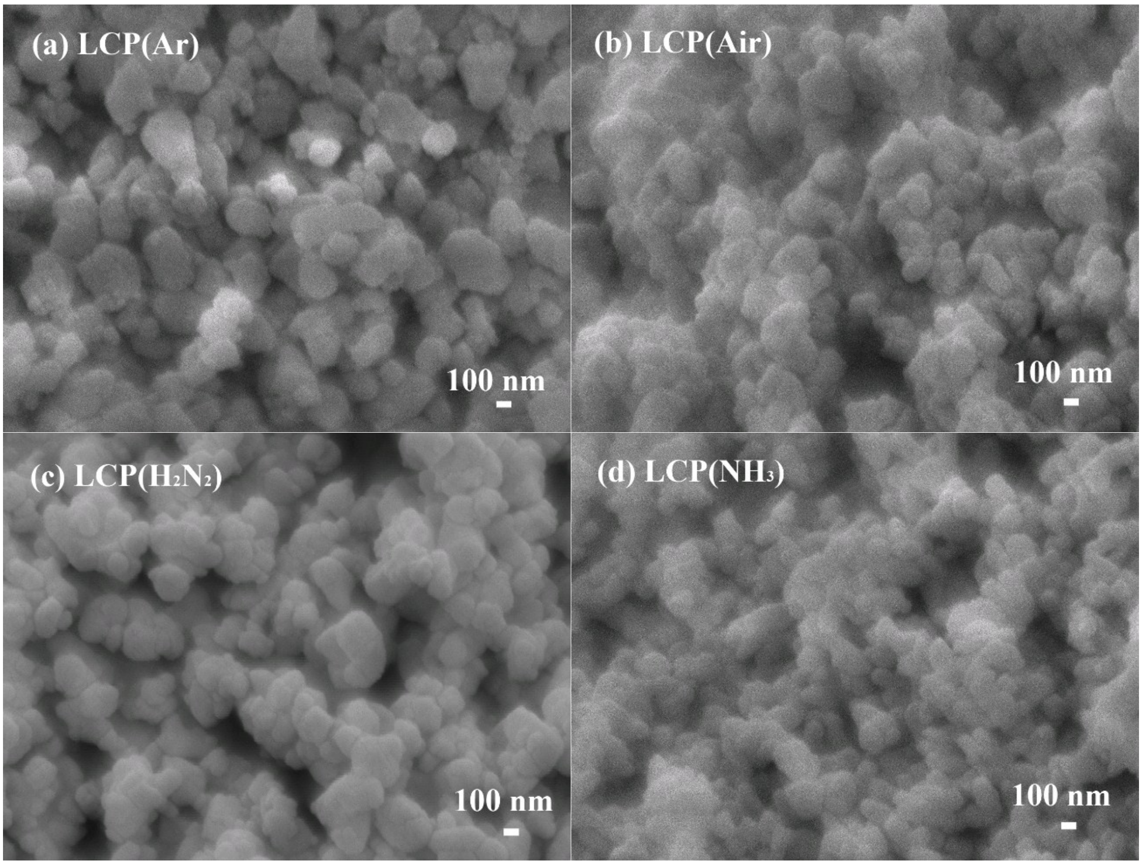

Figure S3 SEM images of (a) LCP(Ar), (b) LCP(Air), (c) LCP(H_2N_2) and (d) LCP(NH_3) (scale bar = 100 nm). Sample labels are explained in Scheme 1.

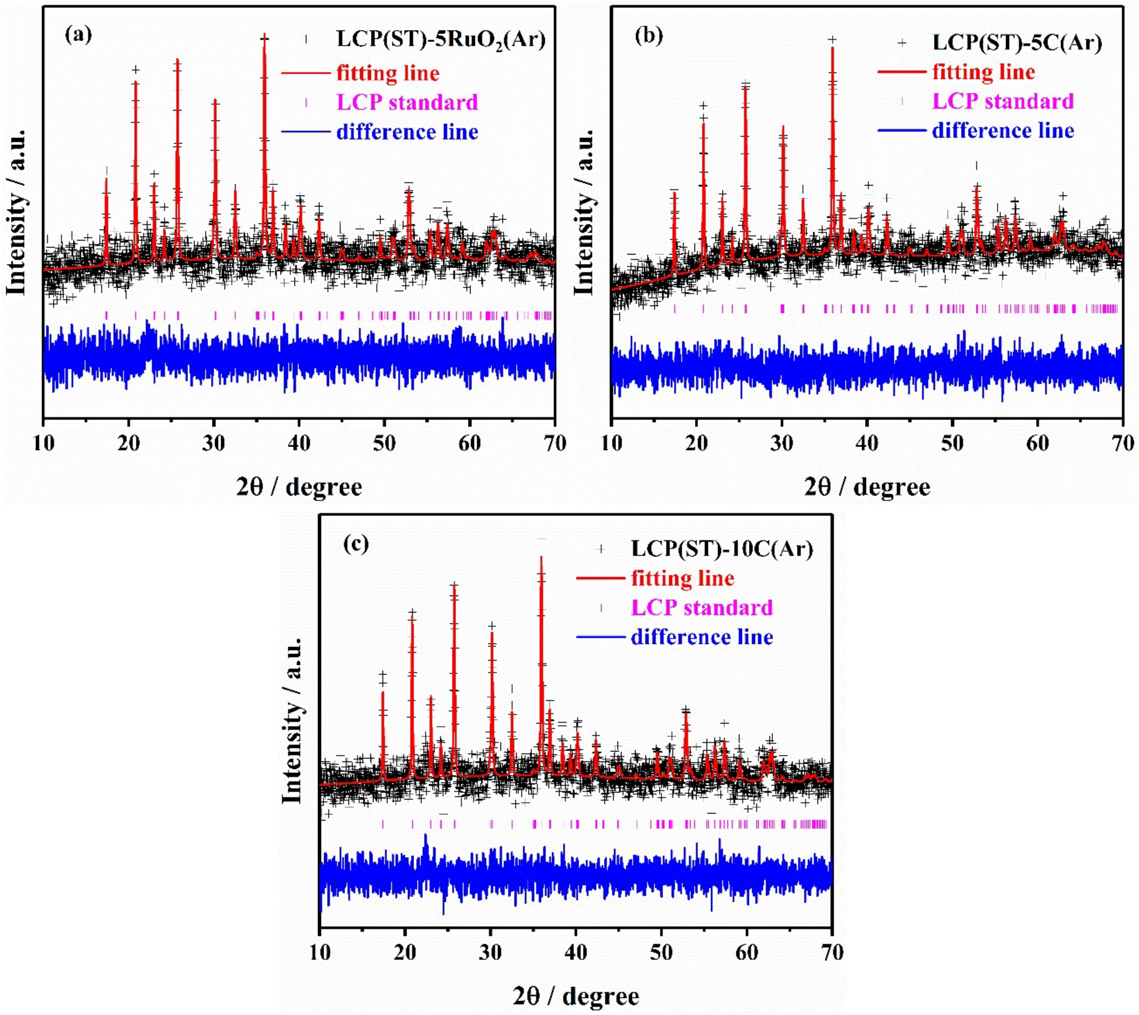

Figure S4 Rietveld fits to the XRD patterns of (a) LCP(ST)-5RuO$_2$(Ar) (R_{wp} = 2.79% and R_p = 2.22%), (b) LCP(ST)-5C(Ar) (R_{wp} = 2.81% and R_p = 2.23%) and (c) LCP(ST)-10C(Ar) (R_{wp} = 2.67% and R_p = 2.10%) (sample labels explained in Scheme 2). The data points and Rietveld fits are overlaid in black crosses and red lines, respectively. The difference plots are shown in blue. The pink tick marks represent the allowed reflection positions for LiCoPO$_4$ with space group Pnma.

Table S2 Lattice parameters obtained from the Rietveld fits to the XRD patterns of the RuO$_2$ and C (5 or 10 wt.%) coated LiCoPO$_4$ samples (labels explained in Scheme 2).

Sample	a / Å	b / Å	c / Å
LCP(ST)-5RuO$_2$(Ar)	10.2048(11)	5.9269(5)	4.6995(5)
LCP(ST)-5C(Ar)	10.2064(16)	5.9277(9)	4.7007(9)
LCP(ST)-10C(Ar)	10.2076(10)	5.9283(5)	4.7015(5)

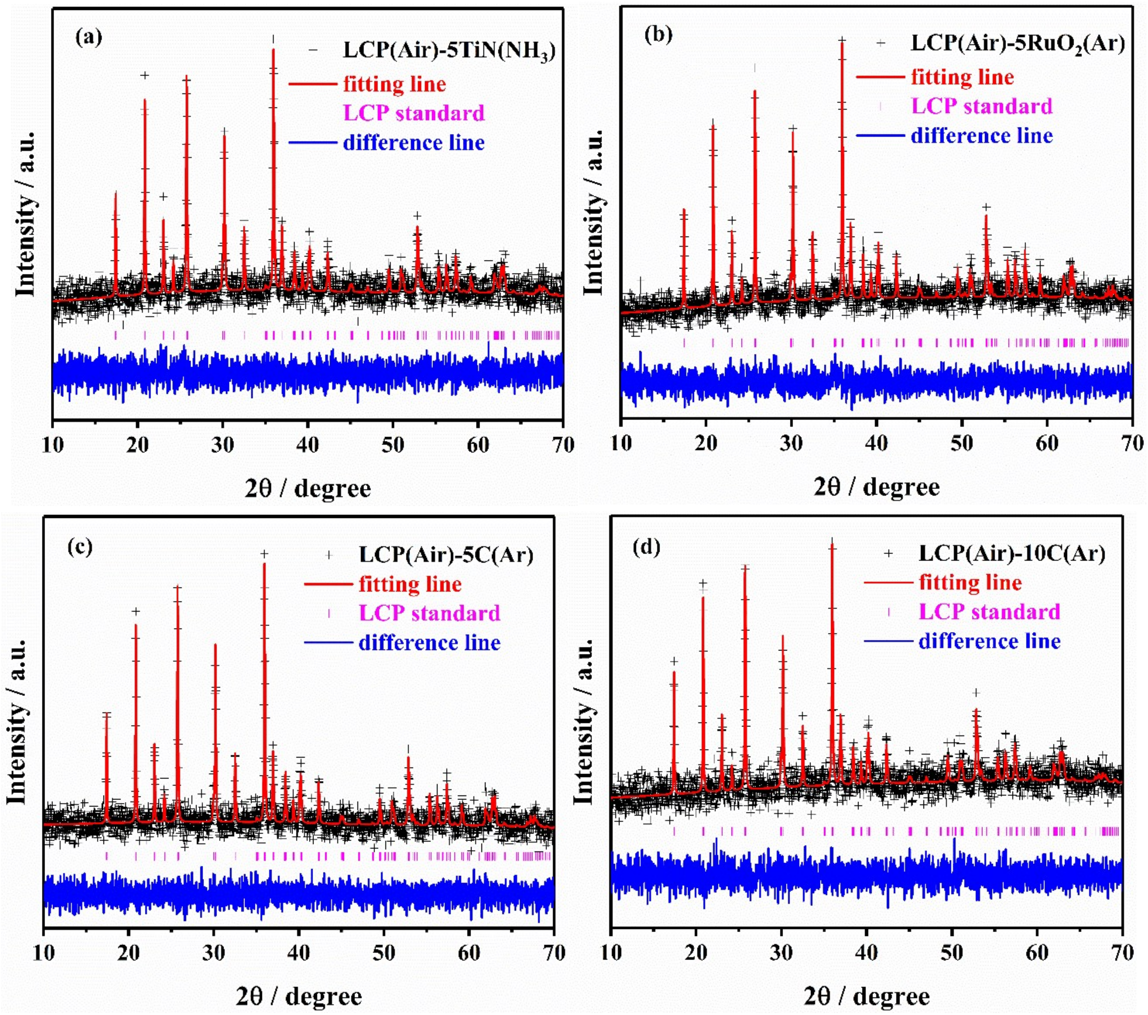

Figure S5 Rietveld fits to the XRD patterns of (a) LCP(Air)-5TiN(NH_3) (R_{wp} = 2.89% and R_p = 2.32%), (b) LCP(Air)-5RuO_2(Ar) (R_{wp} = 2.91% and R_p = 2.31%), (c) LCP(Air)-5C(Ar) (R_{wp} = 2.68% and R_p = 2.14%) and (d) LCP(Air)-10C(Ar) (R_{wp} = 2.80% and R_p = 2.23%) (sample labels explained in Scheme 2). The data points and Rietveld fits are overlaid in black crosses and red lines, respectively. The difference plots are shown in blue. The pink tick marks represent the allowed reflection positions for $LiCoPO_4$ with space group Pnma.

Table S3 Lattice parameters obtained from the Rietveld fits to the XRD patterns of the TiN, RuO_2 and C (5 or 10 wt.%) coated $LiCoPO_4$ samples (labels explained in Scheme 2).

Sample	a / Å	b / Å	c / Å
LCP(Air)-5TiN(NH_3)	10.2053(8)	5.9268(4)	4.7005(4)
LCP(Air)-5RuO_2(Ar)	10.2054(6)	5.9235(3)	4.7003(3)
LCP(Air)-5C(Ar)	10.2039(6)	5.9256(3)	4.7007(3)
LCP(Air)-10C(Ar)	10.2056(8)	5.9268(4)	4.7008(4)

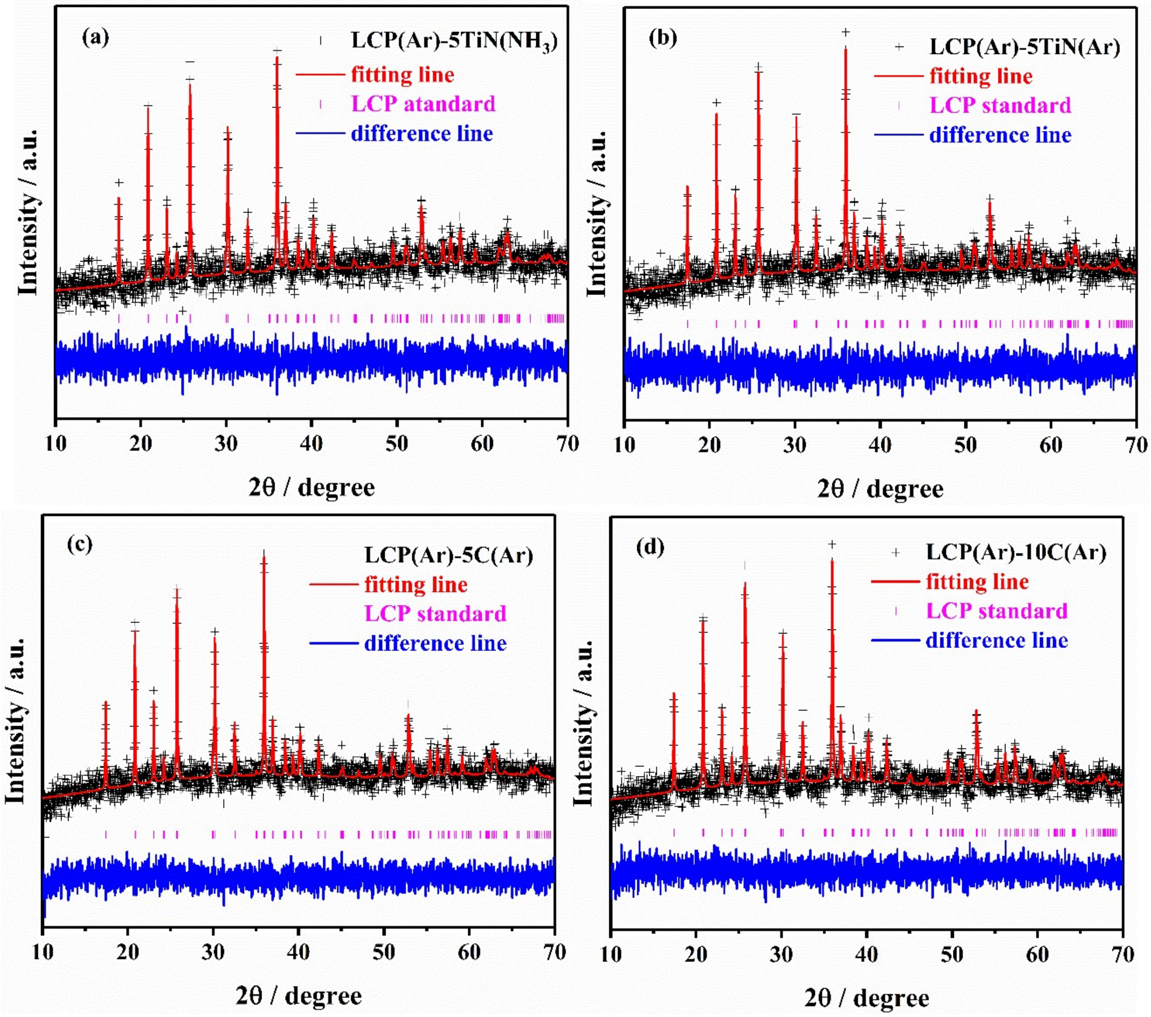

Figure S6 Rietveld fits to the XRD patterns of (a) LCP(Ar)-5TiN(NH_3) (R_{wp} = 2.96% and R_p = 2.36%), (b) LCP(Ar)-5TiN(Ar) (R_{wp} = 2.98% and R_p = 2.37%), (c) LCP(Ar)-5C(Ar) (R_{wp} = 2.84% and R_p = 2.24%) and (d) LCP(Ar)-10C(Ar) (R_{wp} = 2.73% and R_p = 2.19%) (sample labels explained in Scheme 2). The data points and Rietveld fits are overlaid in black crosses and red lines, respectively. The difference plots are shown in blue. The pink tick marks represent the allowed reflection positions for $LiCoPO_4$ with space group Pnma.

Table S4 Lattice parameters obtained from the Rietveld fits to the XRD patterns of the TiN and C (5 or 10 wt.%) coated $LiCoPO_4$ samples (labels explained in Scheme 2).

Sample	a / Å	b / Å	c / Å
LCP(Ar)-5TiN(NH_3)	10.2084(10)	5.9287(5)	4.7018(4)
LCP(Ar)-5TiN(Ar)	10.2029(9)	5.9252(4)	4.7010(4)
LCP(Ar)-5C(Ar)	10.2075(7)	5.9255(4)	4.7005(4)
LCP(Ar)-10C(Ar)	10.2106(8)	5.9286(4)	4.7016(4)

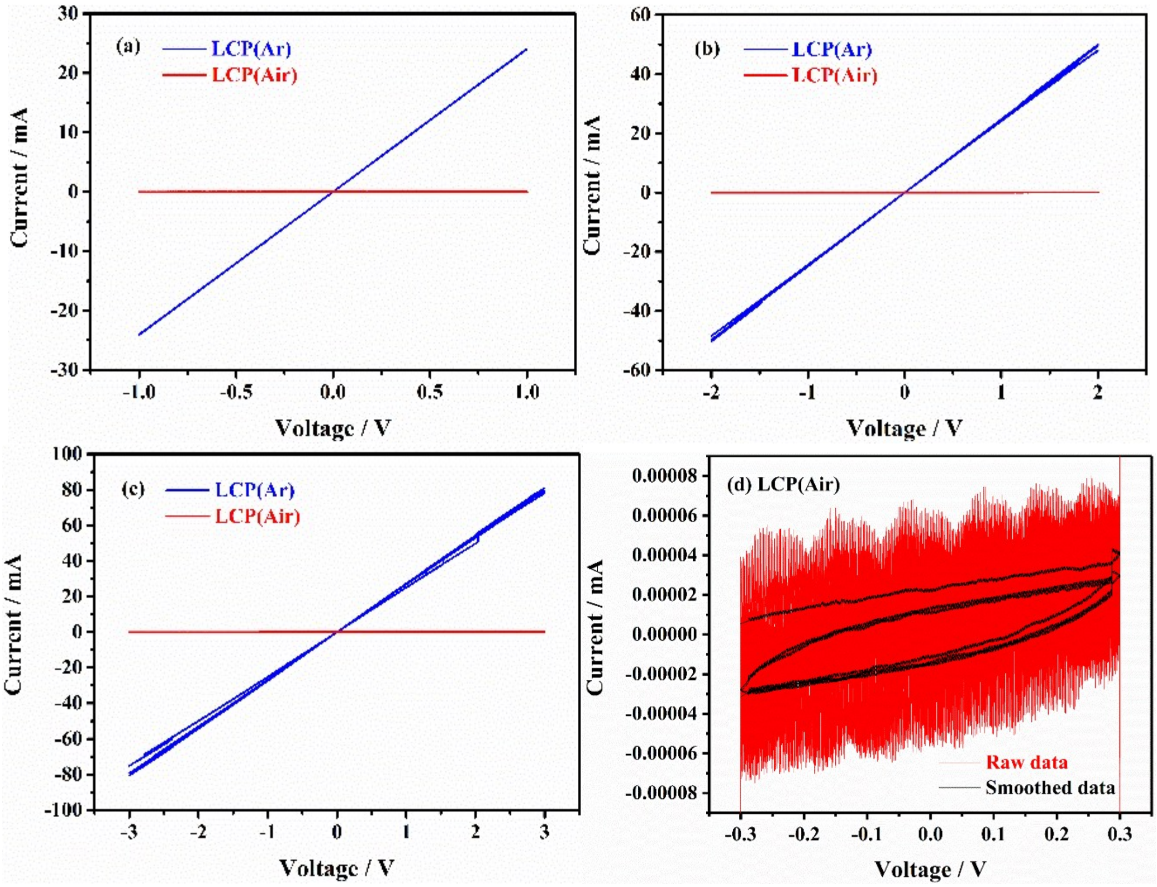

Figure S7 Current-voltage plots (3 cycles each) for $LiCoPO_4$ samples fired in Ar and air, respectively, cycling at scanning rate of 20 mV s^{-1}, showing the ohmic behaviour of the samples (labels explained in Scheme 1).

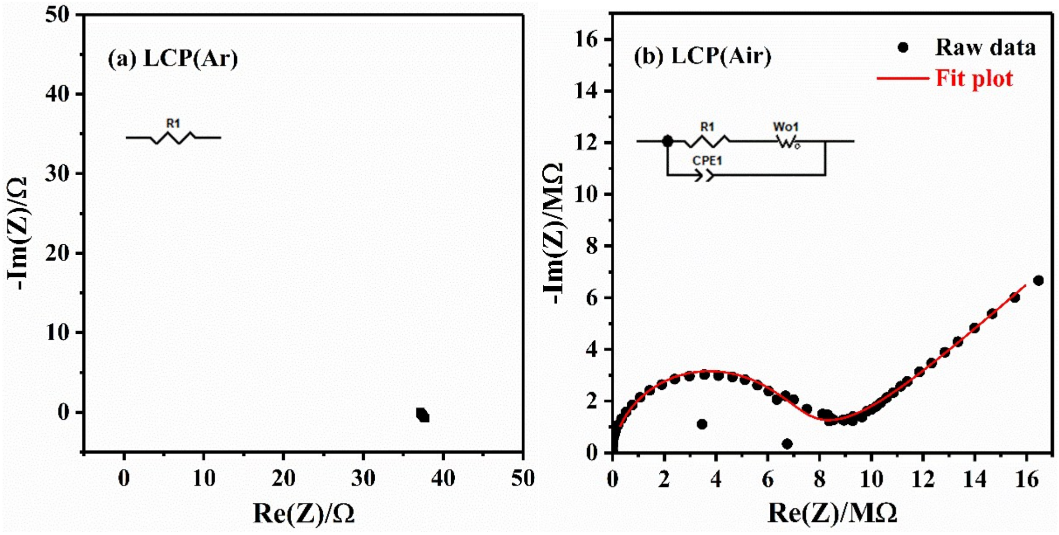

Figure S8 Electrochemical impedance spectroscopy (Nyquist plots) of (a) LCP(Ar) and (b) LCP(Air) samples, collected at 500 mV in the frequency range of 0.1 Hz to 200 kHz. The insets show equivalent circuits used to fit the data (labels explained in Scheme 1).

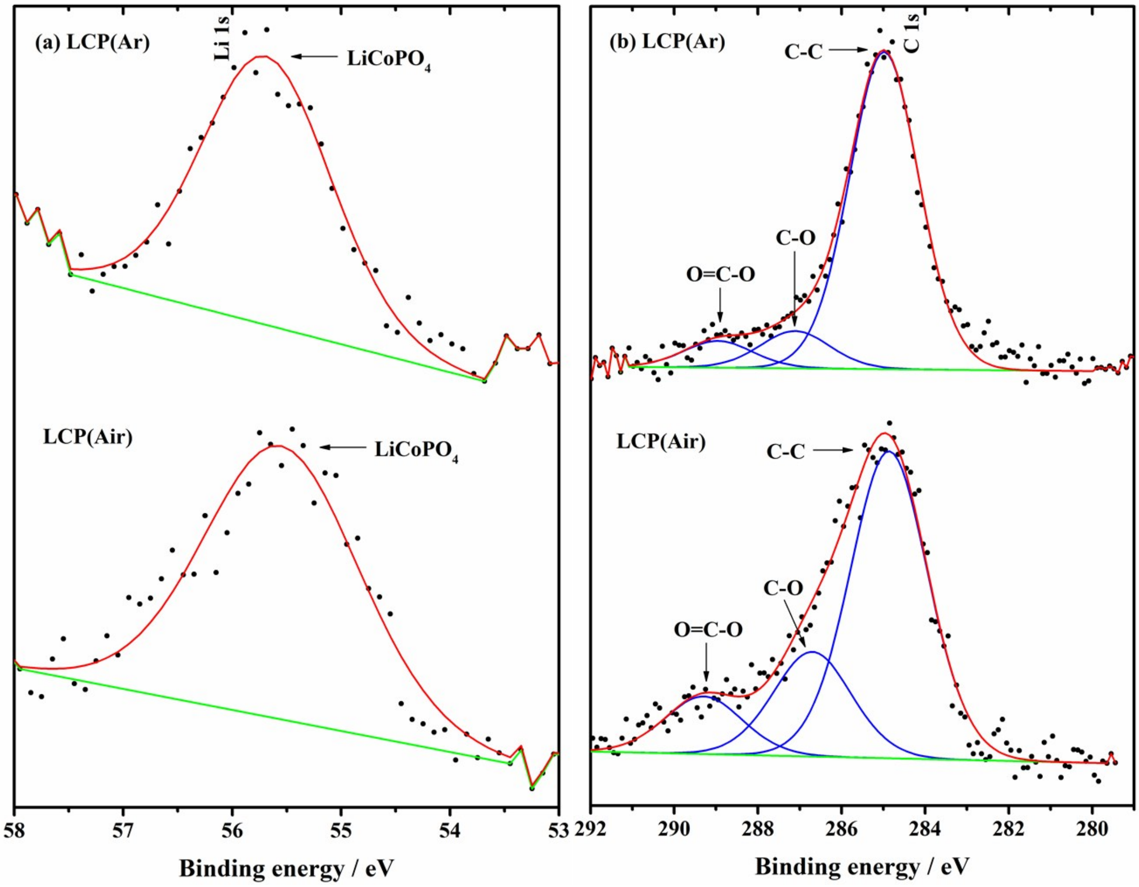

Figure S9 Li 1s and C 1s XPS spectra of $LiCoPO_4$ samples fired in Ar and air, respectively (labels explained in Scheme 1). The data points and enveloped fitting plot are overlaid in black dots and a red line, respectively. The fitting peaks and background are shown in blue and green, respectively.
